# Anoctamin 3: A Possible Link between Cluster Headache and Ca^2+^ Signaling

**DOI:** 10.3390/brainsci9080184

**Published:** 2019-07-30

**Authors:** Caroline Ran, Carmen Fourier, Donia Arafa, Franziska Liesecke, Christina Sjöstrand, Elisabet Waldenlind, Anna Steinberg, Andrea Carmine Belin

**Affiliations:** 1 Department of Neuroscience, Karolinska Institutet, Biomedicum D7, Solnavägen 9, 171 65 Stockholm, Sweden; 2 Department of Clinical Neuroscience, Karolinska University Hospital, Tomtebodavägen 18A, 171 77 Stockholm, Sweden; 3 Division of Neurology, Karolinska University Hospital, Eugeniavägen 3, 171 76 Solna, Sweden

**Keywords:** rs1531394, ANO3, TMEM16C, C11ORF25, ITGAL, fibroblast, expression, reference gene

## Abstract

Cluster headache is a severe primary headache characterized by extremely painful attacks of unilateral headache. Verapamil is commonly used as a prophylactic treatment with good effect. In order to search for new pathways involved in the pathophysiology of cluster headache, we analyzed genetic variants that were previously linked to verapamil response in migraine in a Swedish cluster headache case-control sample. We used TaqMan qPCR for genetic screening and performed a gene expression analysis on associated genes in patient-derived fibroblasts, and further investigated which reference genes were suitable for analysis in fibroblasts from cluster headache patients. We discovered a significant association between *anoctamin 3*, a gene encoding a calcium-activated ion channel, and cluster headache. The association was not dependent on verapamil treatment since the associated variant, rs1531394, was also overrepresented in patients not using verapamil. No difference was found in the *anoctamin 3* gene expression between controls and patients. Also, we determined that *TBP*, *IPO8* and *PDHB* were suitable reference genes in cluster headache fibroblasts. This finding is the first report of an association between a variant in a gene encoding an ion-channel and cluster headache, and the first significant genetic evidence of calcium involvement in cluster headache pathophysiology.

## 1. Introduction

Cluster headache (CH) is a trigeminal autonomic cephalalgia; patients typically suffer from recurrent unilateral headaches of extremely severe pain, accompanied by unilateral autonomic symptoms and restlessness [1]. The most characteristic feature of CH is the intensity of the pain, which is reported to be unparalleled by any other human disorder or physiological condition [2]. The symptoms occur in active periods, followed by asymptomatic remission periods in episodic patients. In contrast, around 15% of the patients are classified as chronic and have no, or very short, remission periods [1]. Another striking singularity of CH is that two thirds of the patients report that their attacks appear with a circadian pattern [3,4]. In particular, the headaches tend to occur at night when the patient is asleep. Around 1 in 1000 individuals are estimated to suffer from CH globally, and there is a clear male predominance [5]. CH pathophysiology is only partly understood, but probably involves both central and peripheral parts of the nervous system. There is evidence of activation of the trigeminal nerve by calcitonin gene-related peptide (CGRP) release [6,7]. This probably entrains the activation of the trigeminal autonomic reflex responsible for the autonomic symptoms experienced during an attack [1]. The hypothalamus is also reported to be activated during a CH attack, a possible explanation for the rhythmic pattern with which the symptoms occur during active periods [8,9]. The cause of CH is currently unknown, but several studies suggest that genetic factors contribute to the risk of developing the disorder [10]. Genetic associations with CH have today been reported for several candidate genes [11,12,13,14,15,16]. The two most extensively studied genes in relation to CH are *clock circadian regulator* (*CLOCK*) and *hypocretin receptor 2* (*HCRTR2*) which are both linked to diurnal rhythm and sleep; however, results are not consistent between studies [17,18,19,20,21]. As yet, there has only been one small study reported using a genome wide association approach. Three new loci were identified, whereof *pituitary adenylate cyclase-activating polypeptide (PACAP) type 1 receptor* can be considered of particular interest [22,23]. None of the reported loci were replicated in an independent sample [24].

Around half of the CH patients in our Swedish biobank use prophylactic treatment (51%) during active cluster periods, and the most commonly used preventive drug is verapamil (34%) [4]. Verapamil is an L-type calcium (Ca^2+^) channel antagonist, which inhibits the influx of Ca^2+^ and therefore affects muscle contraction. Due to its potent vasodilating properties and its effect on contractility and conduction of the myocardium, verapamil is primarily used to treat cardiovascular disorders, such as hypertension, angina and tachycardia, but is also used as a preventive treatment for CH, and to some extent for migraine [25,26,27].

The mechanism of action for verapamil in headache prevention is yet to be elucidated. By comparison to cardiovascular disease, there is a striking difference in effective dosage, specifically for CH patients [25], which might be indicative of a different mode of action or target. Verapamil is not a selective antagonist for L-type Ca^2+^ channels, which is the primary target for its cardiovascular usage. In addition, verapamil also binds to T-type Ca^2+^ channels [28,29]. Results from rodent in vitro experiments further suggest that N-type as well as P/Q-type Ca^2+^ channels might be a target for verapamil [30]. All of these channels are present in nervous tissue, and thus constitute potential targets for a neural mechanism of action of verapamil in CH, as well as a vascular mechanism. Experiments on rodents indicate that verapamil has an analgesic effect on certain types of pain-stimuli [31]. Moreover, verapamil has been shown to block potassium channels in vitro [32,33,34]; e.g., hERG (the human Ether-à-go-go-Related Gene) channels present in peripheral tissue, as well as in the central nervous system [35], and TRESK (TWIK-related spinal cord potassium) channels, which are linked to excitability and pain signaling of trigeminal neurons [36]. The potassium channel subfamily *K member 18* (*KCNK18*), which encodes TRESK has been suggested as a candidate gene for migraine [37]. Interestingly, verapamil is a substrate of the P-glycoprotein transporter, a major drug efflux transporter in the blood brain barrier, and therefore verapamil has low bioavailability in the brain [38,39]. Verapamil also inhibits the P-glycoprotein transporter at higher concentrations [40,41,42]. Hitherto, there are no indications that the relatively high doses of verapamil needed for CH prevention, commonly 240–720 mg per day and sometimes higher [25], are sufficiently elevated to inhibit P-glycoprotein transporters, thereby reducing the outward transport of verapamil at the blood brain barrier. Still, these molecular interactions might provide some explanation for the high doses necessary for prophylactic treatment in CH, and a possibility of a central mechanism of action for verapamil in CH prevention.

In a study aiming to correlate genetics to a good response to verapamil in migraine patients, eight genetic variants in seven genes were found to be associated with verapamil-responsive migraine, measured as a reduction in migraine days after three months of verapamil treatment [43]. These seven genes were implicated in a variety of cellular functions, and further studies of their importance to verapamil treatment might increase the understanding of the mechanism of action for verapamil treatment in migraine. Since the effect of verapamil treatment on CH is superior to the effect on migraine, we decided to investigate these markers as potential genetic risk- or protective factors for CH. Based on the strength of association to a successful verapamil treatment of migraine [43], and a potential role for the corresponding gene in CH pathophysiology, we selected four markers in four different genes; rs17844444 in *protocadherin β6* (*PCDHB6*), rs10882386 in *phospholipase C ε1* (*PLCE1*), rs1531394 in *anoctamin 3* (*ANO3*), and rs2230433 in *α subunit* of *integrin lymphocyte function associated antigen-1* (*ITGAL*). All of these genes have an indirect connection to Ca^2+^ or Ca^2+^ signaling, which is compelling in view of the effect of verapamil on Ca^2+^ influx. For this study we used biological tissue from patients and controls in our extensive Swedish CH biobank, which has been thoroughly characterized previously [4].

## 2. Materials and Methods

### 2.1. Patients and Controls

The case-control material consisted of 628 CH patients and 586 controls (Table 1). Informed consent was obtained from all participants. Experiments were approved by the Regional Ethical Review Board in Stockholm, Sweden (diary number 2014/656-31/4) and carried out according to the rules of the Declaration of Helsinki. Patients were asked to provide personal and clinical information through a questionnaire. CH diagnosis, according to the criteria of the International Classification of Headache Disorders, second edition (ICHD-II) [44], was confirmed by a neurologist. Demographic details for the selection of patients analyzed here are presented in Table 1. Anonymous healthy blood donors were included as control subjects. Genomic DNA was isolated from blood samples according to standard protocols using the Gentra Puregene Blood Kit (QIAGEN, Hilden, Germany). The DNA was quantified and assessed for purity by spectrophotometry with a NanoDrop^TM^ system (Thermo Fisher Scientific, Waltham, MA, USA).

### 2.2. Genotyping

DNA samples from patients and controls were genotyped for four single nucleotide polymorphisms (SNPs), rs17844444, rs10882386, rs1531394, and rs2230433; detailed information on the SNPs can be found in Table 2. SNP genotypes were determined by TaqMan^®^ quantitative real-time PCR (qPCR) on a 7500 Fast instrument (Applied Biosystems, Foster City, CA, USA). The cycling conditions were as follows: 60 °C for 1 min, 95 °C for 10 min, 45–55 cycles at 95 °C followed by 1 min at 60 °C. We used TaqMan^®^ Genotyping Master Mix and commercially available TaqMan^®^ SNP genotyping assays (Applied Biosystems); see Table 2. All four assays had a call rate higher than 98%.

### 2.3. Establishment of Fibroblast Cell Lines

A superficial skin biopsy around 2 × 5 mm in size was taken on the inside of the arm at the site of *musculus biceps brachii*. The procedure was performed under local anesthesia after informed consent. Tissue was cut into smaller pieces and put in culture in a 35-mm Petri dish (Corning, New York, NY, USA) under a coverslip according to the protocol described by Takashima et al. [45]. After a few weeks in a humidified 37 °C, 5% CO_2_ incubator, fibroblasts started to grow along the edges of the tissue; during this time medium was changed 2–3 times per week (DMEM GlutaMAX^TM^ medium with 25 mM D-Glucose and 1 mM sodium pyruvate, supplemented with 13% fetal bovine serum (FBS), 10 mM HEPES and 1% antibiotic-antimycotic (10,000 U/mL penicillin, 10,000 mg/mL streptomycin, and 25 mg/mL amphotericin B), (Invitrogen, Carlsbad, CA, USA)). When cells were confluent, they were transferred to 25-cm^2^ tissue flasks, passaged once to 75-cm^2^ flasks, and then stored at −150 °C in FBS with 10% DMSO (Sigma-Aldrich, St. Louis, MO, United States).

### 2.4. cDNA Preparation

We used primary fibroblast cell lines established from 12 CH patients and 9 control subjects for gene expression analysis. Prior to harvesting, the cells were passaged one time, cultured in 75-cm^2^ flasks until 80% confluency, then starved for 24 h and subjected to a serum shock according to a protocol from Johansson et al., in order to reset the circadian clock [46]. Cells were harvested 6 h after serum shock, and cell pellets were frozen at −150 °C until further use. We used RNeasy Mini Kit (QIAGEN) to obtain RNA from frozen cell pellets according to the manufacturer’s instructions. After RNA quantification using a NanoDrop^TM^ system (Thermo Fisher Scientific), cDNA was prepared from 1 µg of RNA template using the QuantiTect Reverse Transcription Kit (QIAGEN), also according to the provided protocol.

### 2.5. Reverse Transcription qPCR (qRT-PCR)

Primers (Thermo Scientific) were designed with Primer3 software [47,48], verified with Mfold [49], and NCBI nucleotide blast (https://blast.ncbi.nlm.nih.gov/Blast.cgi) tools, for primer sequences refer to Table 3. qRT-PCR was performed on a 7500 Fast instrument (Applied Biosystems), using a standard program for relative quantification: 50 cycles of denaturation at 95 °C for 15 s and annealing/extension at 60 °C for 1 min, terminated by a melting curve.

In order to select a suitable reference gene for gene expression normalization in human primary fibroblasts from CH patients, we first assessed the expression of five reference genes: *GAPDH*, *IPO8, PPIA*, *PDHB* and *TBP* in 12 CH patients and 8 controls. qRT-PCR was performed with Power SYBR^®^ Green PCR Master Mix (Thermo Fisher Scientific), 200 ng cDNA and 0.3 µM of each primer in each reaction. For the relative quantification of *ANO3* mRNA, we used 300 ng cDNA from 11 CH patients and 9 controls, Power SYBR^®^ Green PCR Master Mix, and 0.5 µM primers for target gene *ANO3*, with reference genes *IPO8* and *TBP*.

### 2.6. Statistical Analysis

Power calculation was performed using the PS Power and Sample Size Calculator v3.0 [50], and was based on minor allele frequencies (MAF) reported for European populations in the NCBI dbSNP database (https://www.ncbi.nlm.nih.gov/projects/SNP/). Using our sample size and an MAF of 0.15, an association with an odds ratio (OR) <0.61 or >1.53 would be detected with 80% power, and with a MAF of 0.3 the corresponding OR would be <0.69 or >1.41. Genotypes were determined using the 7500 software v2.3 provided with the 7500 Fast instrument (Applied Biosystems). Genetic association was analyzed using a two-tailed chi-squared (χ^2^) test or a Fisher’s exact test in GraphPad Prism v5.04 (GraphPad Softwares Inc, La Jolla, CA, USA). We used the Šidák correction for multiple testing, adjusting the threshold for significance to α_SID_ = 0.013. The Hardy-Weinberg equilibrium (HWE) was assessed in both patients and controls using the OEGE HWE calculator [51]. Control individuals were in HWE for all four SNPs, but CH patients exceeded the threshold for HWE χ^2^ > 3.84 (*p* = 0.05), for rs1744444—χ^2^ = 5.86, and rs1531394—χ^2^ = 9.59. The other two SNPs, rs10882386 and rs2230433, were in HWE in CH patients as well.

In silico analysis of associated SNPs was performed with online computational tools—Mfold and SNP2TFBS [49,52].

Reference genes were statistically analyzed using GraphPad Prism v5.04, verifying means and variances, and further compared using Normfinder and geNorm [53,54]. *TBP* and *IPO8* were determined to be the most stable reference genes for CH in fibroblasts.

*ANO3* mRNA levels were normalized to *IPO8* and *TBP* and to one randomly selected control sample in the 7500 software v2.3 provided with the 7500 Fast instrument. One patient was removed from the analysis due to high variability between technical replicates. Data was analyzed with Grubb’s test to exclude outliers (Z > 2.4, significance level 0.05); none were identified. We performed a log_2_-transformation, due to non-normal distribution according to the D’Agostino and Pearson omnibus normality test, and thereafter analyzed the data with Student’s t-test, one-way ANOVA, and linear regression; significance level was set to 0.05, and two-tailed p-values were used.

## 3. Results

### 3.1. Genetic Analysis

586 control subjects and 628 individuals with CH from a Swedish biobank were genotyped for four different markers (Table 2) which are suggested to be linked to response to verapamil in migraine. Genotype and allele frequencies for these markers and the results of the analysis are presented in Table 4. One of the variants, rs1531394, located in *ANO3*, was discovered to be more common amongst CH patients. In particular, individuals carrying two copies of the minor allele were clearly overrepresented in the CH group: 19.45% as compared to 13.75% in controls; this difference was significant (*p* = 0.0097) (Table 4). Further analysis showed that the effect was strongest under a recessive model with an OR of 1.52, resulting in *p* = 0.0086 (Table 5). Statistical analysis also revealed that rs1531394 was not in HWE in patients. In contrast to the *ANO3* variant, the wild type genotype GG of rs2230433 in *ITGAL* seemed to be more common in the CH patients, suggesting a protective effect of the minor allele (Table 4). Statistical analysis of the data showed only a trend for association between the C allele and CH (*p* = 0.066), and the genotype analysis showed no significance. However, a secondary analysis under a dominant model confirmed the trend for a protective association between rs2230433 and CH; OR 0.79, 95% confidence interval (CI) 0.63–0.99, *p* = 0.043 (data not shown). The other two genetic variants, rs17844444 and rs10882386, were equally distributed in the patient and control groups.

About one third of the CH patients (30.4% = 191 individuals) used verapamil as a prophylactic treatment for CH. We performed a stratified analysis under a recessive model, comparing patients using verapamil and those not using verapamil separately with controls. We found an association between rs1531394 and CH in both groups together and separately (Table 5). The risk genotype AA was slightly more common in the subgroup of patients using verapamil compared to non-users: 20.94% vs. 18.81%.

**Figure A1:**
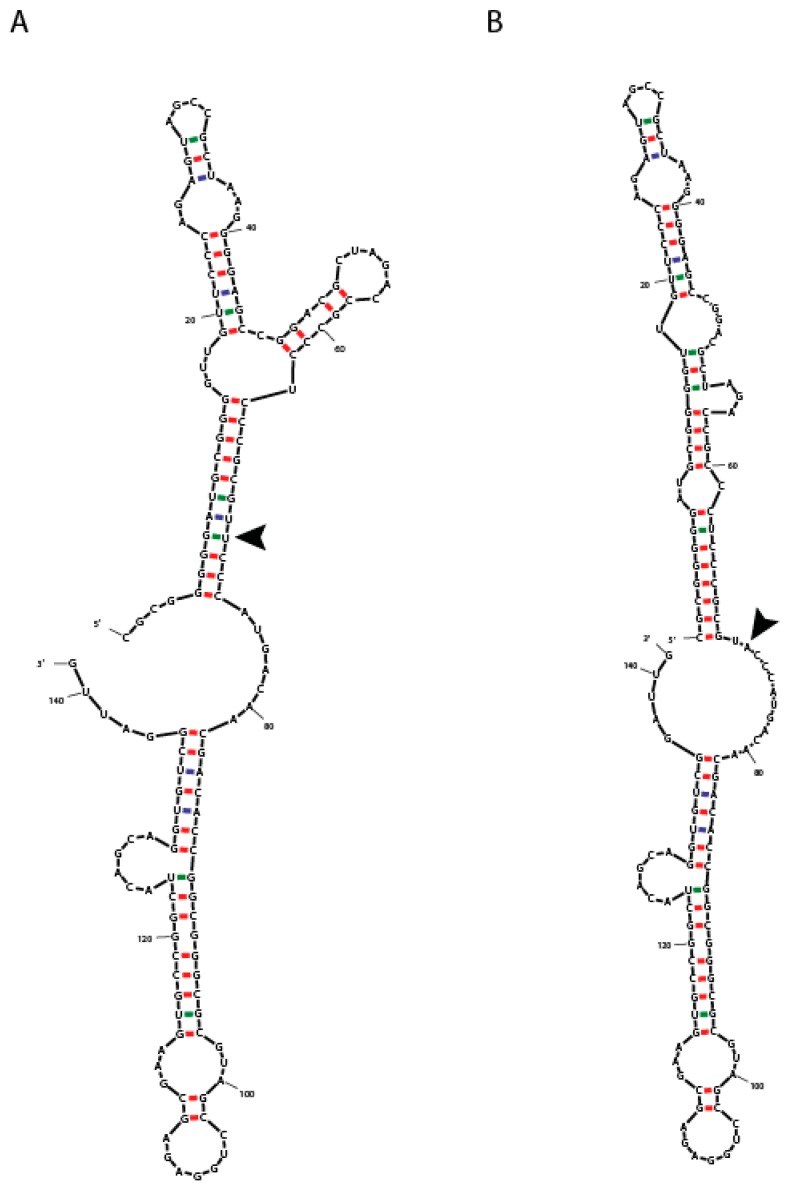
Circle graph of mRNA fragments with the T or A allele at SNP rs1531394. Mfold was used to analyze the effect of rs1531394 on the predicted *ANO3* mRNA folding of 141 bp partial sequence: rs1531394 is indicated by an arrowhead with the T allele in (**A**), and A allele in (**B**).

In silico analysis using the Mfold software to analyze the folding of a segment of mRNA (140 bp) surrounding the disease-associated SNP revealed that an mRNA transcript with the minor allele (A) adapts slightly different secondary structures than the wild type fragment (Figure A1). There was a 5.5% increase of Gibbs energy required to fold the minor allele-containing fragment, as compared to the wild type. As the rs1531394 variant is located in the 5′UTR of the sequence (Table 2); it can potentially affect interactions with transcription factors and other binding proteins. We analyzed the SNP for overlap with transcription factor binding sites using the SNP2TFBS online computational tool and found that rs1531394 potentially affects binding of transcription factor RFX2, which was predicted to bind to the *ANO3* promoter. The position weight matrix (PMW) score was lower for the minor A allele than the T allele, indicating that the sequence with the wild type allele has a better match with the transcription factor binding motif than the disease-associated allele.

### 3.2. Formal Selection of Reference Genes for Studying CH in Fibroblasts

**Figure 1:**
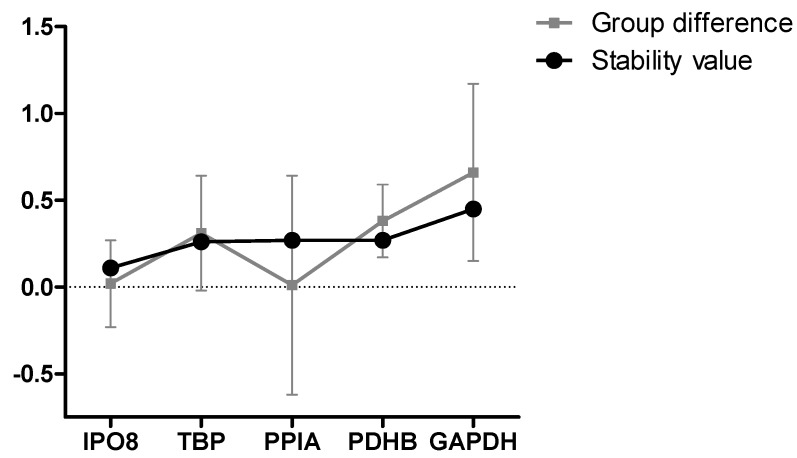
Stability of mRNA expression in fibroblast cell lines from cluster headache (CH) cases and controls. The stability of mRNA expression of five reference genes was assessed in primary fibroblast cell lines from 8 healthy control subjects and 12 CH patients. Comparative analysis to determine the gene with the most stable expression was performed using the software Normfinder [53]. Gene names are listed on the *x*-axis. *IPO8* was the most stable reference gene in these conditions, presenting both high stability (stability value close to zero), and a small difference in mRNA expression (group difference close to zero), with low standard deviation (error bars) between the two groups.

We performed an initial experiment to determine which reference genes were best suited to assess gene expression in primary fibroblast cell lines from CH patients. Five established reference genes were selected for the trial: *GAPDH*, *IPO8, PPIA*, *PDHB,* and *TBP*. Data was first analyzed to assess that means and variances did not differ between cases and controls; this was true for all genes except *GAPDH,* which was more abundant in patients (*t* = 2.333, *p* = 0.0315). Reference genes were then ranked using two different programs, which both showed that *TBP* and *IPO8* were the most stable genes in our experimental setting. In Figure 1, reference genes are ranked with Normfinder based on their intergroup variability (stability) and their intragroup variability (difference between cases and controls). Results from the geNorm analysis, which uses a different algorithm to assess stability, differed slightly from Normfinder by ranking *PDHB* as more stable than *PPIA* (data not shown) [53,54].

### 3.3. Gene Expression Analysis of ANO3 and ITGAL

**Figure 2:**
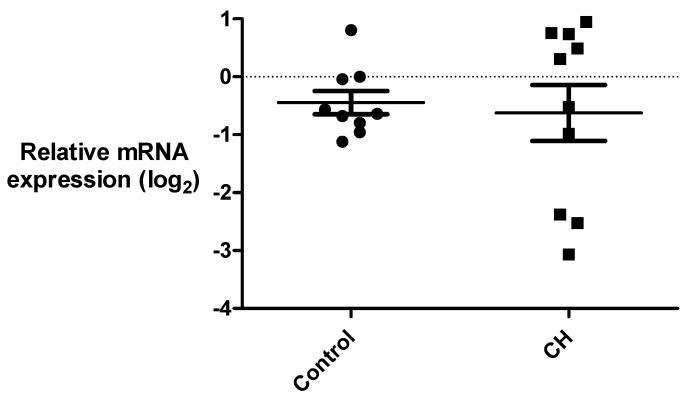
*ANO3* mRNA levels in cluster headache (CH) patients and controls. Relative mRNA levels of *ANO3* in primary fibroblast cell lines from 9 control individuals and 10 CH patients. mRNA levels were normalized to reference genes *TBP* and *IPO8*, and to a control sample. Relative expression levels were log_2_-transformed due to non-normal distribution of the data, and analyzed with Student’s t-test, *p*-value = 0.75.

We further examined the expression of the two most promising candidate genes from the association analysis, *ANO3* and *ITGAL*, using primary fibroblast cell lines derived from a subset of patients and controls. In accordance with what is reported in public databases, we detected low *ANO3* mRNA expression in fibroblasts. We performed a relative quantification of *ANO3*; mRNA levels were normalized and then compared between cases and controls. We observed no difference in *ANO3* gene expression between the two groups (*p* = 0.75), but mRNA levels in the patient group were more spread than in the control group, and the variances were significantly different between cases and controls (*F* = 6.55, *p* = 0.015); see Figure 2. Therefore, we analyzed the expression data according to several other parameters, which were clinical subtype, gender, age, rs1531394 genotype, and verapamil use, but none of those parameters could be correlated to the high variability in the patient group (data not shown). *ITGAL* mRNA was expressed at too low levels in fibroblasts to perform a reliable analysis.

## 4. Discussion

This analysis on four genetic markers that were originally linked to verapamil response in migraine permitted us to identify two new candidate genes for CH: *ANO3,* and possibly *ITGAL*. We found an association between rs1531394 in *ANO3* and an increased risk for CH. Interestingly, the rs1531394 variant was found to associate to CH both in patients using verapamil and patients not using verapamil, implying that the association does not depend on verapamil use, but is attributed solely to CH. One limitation of our study is that we did not have information as to why 69.6% of our patients did not use verapamil. Some of these patients might never have used preventive treatment, discontinued their treatment because of adverse effects, or were in remission and not in need of prophylactic treatment. That information could potentially reveal an association also to verapamil responsiveness which would be masked in the present study. This type of data could in the future have clinical impact, as a genetic test might precede and optimize the choice of medication.

A general limitation for candidate gene studies in CH have so far been small sample sizes (*n* = 100–200) and heterogeneous study designs, which have led to inconclusive results in the past. In addition, it is of great importance to validate our findings with other large independent case-control studies before drawing definite conclusions on the involvement of *ANO3* in CH pathophysiology. *ANO3* is a large gene on chromosome 11 comprising 27 exons, and further investigations of other genetic variants within the same gene in relation to CH are warranted.

The variant studied here resides in the 5′ UTR region of the gene sequence, and therefore has a potentially regulatory role. Specifically, the rs1531394 SNP is localized in a potential binding site for transcription factor RFX2. Our data on *ANO3* in CH patients showed no difference in expression levels between patients and controls in fibroblasts, but a large variability in the patient group that we could not account for in our analysis on subtype, gender, age, or verapamil use. We did not investigate whether exposure to verapamil in vitro affected *ANO3* gene expression; this might be an informative approach in the future. Moreover, gene expression in fibroblasts did not correlate to rs1531394 genotype. That is in contrast to data from the GTEx portal (https://gtexportal.org) suggesting that rs1531394 represents an expression quantitative trait locus (eQTL) in the tibial nerve. Consequently, it would be of great interest to investigate the gene expression of *ANO3* in other human tissues, specifically in nervous tissue, where *ANO3* is expressed abundantly, and likely to have a more prominent role than in skin. The rs1531394 variant might also affect *ANO3* in ways other than gene expression. SNPs in the untranslated regions of a gene can for example affect mRNA stability, as suggested by our in silico analysis, revealing an effect on the secondary structure of the mRNA molecule. Additionally, binding or transport of the transcript might be affected by an untranslated SNP. Moreover, it is possible that the SNP studied by us is not the causative SNP per se, but that it is in linkage disequilibrium with other SNPs representing the true association.

Our findings on *ANO3* are highly interesting, as they represent a molecular indication of Ca^2+^ signaling as a new pathophysiological pathway in CH. Verapamil, and other Ca^2+^ channel antagonists are commonly used in headache prevention, but besides the genetic association between familial hemiplegic migraine (type 1) and *CACNA1A* (*calcium voltage-gated channel subunit alpha1 A*), little is known of the implications of Ca^2+^ in headache. *CACNA1A* mutations have been screened and tested for association with CH, but no significant link has been identified [55,56]. *ANO3* has not been studied previously in relation to CH. The gene encodes a Ca^2+^ activated Cl^-^ channel and was previously discovered to be implicated in craniocervical dystonia [57,58]. Anoctamines are a family of 10 genes, and while the first two subtypes (1 and 2) are fairly well described, the role and function of *ANO3* is still unclear [59]. In vitro experiments suggest that it is an intracellular protein, which is supported by the finding that mutations in *ANO3* probably influence Ca^2+^ signaling by reducing the Ca^2+^ pool of the endoplasmic reticulum [57,60]. Interestingly, *Ano3* knock-out rats display hyperexcitability of neurons residing in dorsal root ganglia, and are hypersensitive to pain [61].

The significance of our findings on the rs2230433 variant in *ITGAL* is more uncertain. There was a trend for association between this marker and CH, suggesting a protective effect, and the gene expression was not possible to evaluate in fibroblasts, due to low expression levels. We believe this gene could be of interest, but the association has to be investigated in a large independent CH cohort prior to drawing any conclusions on its potential involvement in CH.

Gene expression analysis permitted us to formally establish *IPO8, TBP*, and *PDHB* as suitable reference genes for studying CH in fibroblasts. Of the five reference genes that were assessed, *GAPDH* (which is a well-established reference gene) turned out to be the least suitable gene for CH, showing a significant difference in mRNA levels between cases and controls. This in an important finding, and should be considered in future gene expression studies on CH and fibroblasts.

## 5. Conclusions

In this paper, we report the first genetic association between CH and a SNP in *ANO3*, which is a gene encoding a Ca^2+^ activated Cl^−^ channel. This finding is of great interest to the field of CH research, as it suggests Ca^2+^ signaling as a new component of CH pathophysiology.

## Figures and Tables

**Table 1:** Demographic data for patients and controls.

	Controls	CH Patients
**Individuals**	586	628
Average age in years ± SD	n/a	52.1 ± 14.6
Male % (*n*)	60.6 (355)	68.3 (429)
Chronic CH % (*n*)	n/a	10.6 (66)
Average age at onset ± SD	n/a	32.0 ± 13.6
Positive family history % (*n*)	n/a	10.8 ^§^ (61)
Prophylactic verapamil use % (*n*)	n/a	30.4 (191)
Diurnal rhythmicity % (*n*)	n/a	68.0 ^†^ (380)

CH: Cluster headache; SD: standard deviation; n/a: not available/applicable; *n*: number of individuals; ^§/†^ information available for a total number of ^§^ 567 individuals or ^†^ 559 individuals, respectively.

**Table 2:** Analyzed SNPs.

Gene	SNP rs Number (HGVS)	Consequence	TaqMan Assay
*PCDHB6*	rs17844444 (NC_000005.10:g.141152584G>A)	Missense Gly→Asp	C__32960006_10
*PLCE1*	rs10882386 (NC_000010.10:g.95790669G>A)	Unknown (5′UTR)	C___1946626_10
*ANO3*	rs1531394 (NC_000011.10:g.26332096T>A)	Unknown (5′UTR)	C___1616984_10
*ITGAL*	rs2230433 (NC_000016.10:g.30506720G>C)	Missense Arg→Thr	C__11789692_10

SNP: single nucleotide polymorphism; HGVS: Human Genome Variation Society; UTR: untranslated region.

**Table 3:** Primers used for gene expression analysis.

**Gene of Interest**		**Forward Primer 5′-3′**	**Reverse Primer 5′-3′**
Anoctamin 3	*ANO3*	CCTGGAGTTTTGGAAAAGGAGA	CTTGGCTTCAAACTGGGGAC
Integrin Subunit α L	*ITGAL*	CACTCTATGTCAGTTTCACCCC	GTTGTGGTCGTGGATGGAAG
**Reference Gene**		**Forward Primer 5′-3′**	**Reverse Primer 5′-3′**
Glyceraldehyde-3-Phosphate Dehydrogenase	*GAPDH*	AGCCACATCGCTCAGACAC	GCCCAATACGACCAAATCC
Importin 8	*IPO8*	ATTGGAAGAAACCGCGCTTG	TGTGTACACCTCCTGCAGTG
Peptidylprolyl Isomerase A	*PPIA*	GACCCAACACAAATGGTTCC	GGCCTCCACAATATTCATGC
Pyruvate Dehydrogenase E1 β Subunit	*PDHB*	GGTTTCCCATTCAAGACCTG	TGGTTTCCATGTCCATTGGT
TATA-Box Binding Protein	*TBP*	AGGCAACACAGGGAACCTC	TTGCAGCTGCGGTACAATCC

**Table 4:** Statistical analysis of genotypes and alleles.

SNP Name	Genotype/Allele	Control % (*n*)	CH % (*n*)	χ^2^ (*df*)	OR (95% CI)	*p*-Value
**rs17844444**	GG	75.30 (439)	75.41 (466)			
	GA	22.64 (132)	21.52 (133)			
	AA	2.06 (12)	3.07 (19)	1.37 (2)		0.50
	G	86.62 (1010)	86.17 (1065)		1.04	
	A	13.38 (156)	13.84 (171)	0.071 (1)	(1.82–1.31)	0.79
**rs10882386**	GG	55.36 (320)	56.64 (354)			
	GA	39.62 (229)	36.80 (230)			
	AA	5.02 (29)	6.56 (41)	1.94 (2)		0.38
	G	75.17 (869)	75.04 (938)		1.007	
	A	24.83 (287)	24.96 (312)	0.0008 (1)	(0.84–1.21)	0.98
**rs1531394**	TT	37.29 (217)	38.28 (240)			
	TA	48.97 (285)	42.27 (265)			
	AA	13.75 (80)	19.46 (122)	9.28 (2)		0.0097 *
	T	61.77 (719)	59.41 (745)		1.10	
	A	38.23 (445)	40.59 (509)	1.31 (1)	(0.94–1.30)	0.25
**rs2230433**	GG	51.55 (299)	57.44 (359)			
	GC	40.69 (236)	35.52 (222)			
	CC	7.76 (45)	7.04 (44)	4.24 (2)		0.12
	G	71.90 (834)	75.2 (940)		0.84	
	C	28.10 (326)	24.8 (310)	3.38 (1)	(0.70–1.011)	0.066

SNP: single nucleotide polymorphism, CH: cluster headache, χ^2^: chi-squared, *df*: degrees of freedom, OR: odds ratio, CI: confidence interval, * α_SID_ = 0.013 using the Šidák correction for multiple testing.

**Table 5:** Analysis of rs1531394 in patients using verapamil.

Genotype	Control % (*n*)	All CH Patients % (*n*)	CH Using Verapamil % (*n*)	CH not Using Verapamil % (*n*)	OR (95%CI)	*p*-Value
TT + TA	86.25 (502)	80.54 (505)			1.56	
AA	13.75 (80)	19.46 (122)			1.11–2.06	0.0086
**Stratified Analysis**						
TT + TA			79.06 (151)		1.66	
AA			20.94 (40)		1.09–2.53	0.021
TT + TA				81.19 (354)	1.45	
AA				18.81 (82)	1.04–2.04	0.031

CH: cluster headache, OR: odds ratio, CI: confidence interval.
